# *In-situ* microfluidic controlled, low temperature hydrothermal growth of nanoflakes for dye-sensitized solar cells

**DOI:** 10.1038/srep17750

**Published:** 2015-12-03

**Authors:** Chao Zhao, Jia Zhang, Yue Hu, Neil Robertson, Ping An Hu, David Child, Desmond Gibson, Yong Qing Fu

**Affiliations:** 1 Department of Physics and Electrical Engineering, Faculty of Engineering & Environment, Northumbria University, Newcastle upon Tyne, NE1 8ST, UK; 2 Thin Film Centre, Scottish Universities Physics Alliance (SUPA), University of the West of Scotland, Paisley, PA1 2BE, UK; 3 Key Laboratory of Micro-systems and Micro-structures Manufacturing, Ministry of Education, Harbin Institute of Technology, No. 2 YiKuang Street, Harbin, P.R. China, 150080; 4 University of Edinburgh, Joseph Black Building, David Brewster Road, Edinburgh, EH9 3FJ, UK; 5 Institute of Thin Films, Sensors & Imaging, University of the West of Scotland, Paisley, Scottish Universities Physics Alliance, PA1 2BE, UK

## Abstract

In this paper, an *in-situ* microfluidic control unit (MCU) was designed and applied in a hydrothermal synthesis process, which provides an easy way to localize liquid-phase reaction and realize selective synthesis and direct growth of nanostructures as well as their morphology, all in a low-temperature and atmospheric environment. The morphology was controlled through controlling the amount of additivities using the MCU. This achieved a facile fabrication of Al doped ZnO (AZO) nanoflakes vertically grown on flexible polymer substrates with enhanced light scattering and dye loading capabilities. Flexible DSSCs with a significant enhancement (410% compare to ZnO NRs based devices) in power conversion efficiency were obtained using AZO nanoflake photoanodes of 6 μm thick, due to the enhancement in electron mobility and reduction in recombination. This hydrothermal synthesis using the *in-situ* MCU provides an efficient and scalable technique to synthesize controllable nanostructures with characteristics of easy set-up, low energy consumption and low cost.

Solution-processed nanostructures of metal oxides (i.e., TiO_2_, ZnO) show great promise for energy-harvesting and electronic devices due to advantages including low cost, ease of preparation, structure/component design and control^1^,^2^,^3^,^4^. For example, dye sensitized solar cells (DSSCs) use nanostructured metal oxides (e.g., nanoparticles, nanowires, nanotubes and nanoflakes) photoanodes, which provide abundant surface areas for binding absorber (dye molecules), and simultaneously offer a continuous pathway for electron transport with minimal recombination. ZnO has been considered as one of the most promising alternative materials for the conventionally used TiO_2_, due to its advantages including higher bulk electron mobility with a suitable band gap and flexibility in morphology control. One and two dimensional (1&2D) nanostructured photoanodes were recently demonstrated to increase surface area for dye loading, improve electron transport with an efficient charge separation, and serve as light-scattering centers to increase the optical length in the photoanode^5^,^6^,^7^,^8^. Unfortunately, DSSCs based on the 1&2D ZnO nanostructures have achieved limited success. For example, power conversion efficiency (PCE) is still lower than that of nanoparticle based photoanodes^9^,^10^,^11^,^12^,^13^,^14^, because of two major challenges: (i) lower internal surface areas of the 1&2D nanostructures (compared with nanoparticles); (ii) lower electron injection efficiency and poor chemical stability of the ZnO^15^,^16^. As a result, a TiO_2_ layer is often used for modification of ZnO nanostructures to stabilize and improve dye loading capability^17^,^18^.

Optimization of photoanode nanostructures can provide not only highly-accessible large surfaces but also a long-range electronic connectivity, which could meet the critical requirements of high performance DSSCs. For example, the PCE has been increased from 2.3–3.9% (for pure nanosheets/nanoflakes) to 5.41% (for mosaic nanosheets composite structures)^19^,^20^,^21^. Additionally, arrays of controllable nanoflakes (NFs) were reported to improve significantly light trapping and solar cell performance^22^. The benefit of ZnO NFs can be further improved by doping other elements into ZnO. Doping of ZnO with elements of group III-(Al, Ga and In) or group IV (Pb, Sn) has been proven as an efficient method to enhance the electrical properties of the ZnO without deteriorating their optical transmission properties^23^,^24^,^25^. Boron-doped ZnO nanosheet-based photoanodes (~1.5 μm thick) were obtained using electrostatic spray which showed a PCE of 6.75%^26^. Al doped ZnO (AZO) has also been extensively investigated/used in solar harvesting applications because of its benefits of high conductivity, low cost and good optical performance^23^,^27^.

Great effort has been made to develop the AZO NF nanostructures using solution based techniques, such as microwave enhanced hydrothermal methods, electro-chemical and electro spraying technique^28^,^29^,^30^,^31^. However, many challenges are still existed in: (1) realization of controlled-vertically grown crystallized AZO NFs directly on the desired substrates for achieving desirable light trapping properties with improved adhesion and contact resistance; (2) simplification of the synthesis process (i.e., avoiding uses of additives to assemble, mutli-step process, or high temperature process, i.e., ~400 °C or above)^17^,^32^,^33^,^34^,^35^.

In this study, we proposed a unique low-temperature, template-free approach to produce vertically aligned AZO NFs and ZnO NRs/AZO NFs hybrid structures for use as photoanodes in flexible DSSCs, which achieved a PCE value of 4.5% using the film with a thickness less than 10 μm. In particular, this structure has been achieved using an *in-situ* microfluidic control unit (MCU) in the hydrothermal reaction vessel, which is simple and cost-effective (as shown in Fig. 1). The additive reactant solution can be supplied directly to the seeded substrates area in the reaction vessel using a microchannel driven by a syringe pump, which results in a localized reaction at the desired surface rather than in the bulk solution. A localised reaction occurs in the defined area, thus producing a localised nanostructure growth. The fresh precursor chemicals in the bulk solutions are continuously supplied to the reaction area by convective mass transfer due to temperature gradient; and the additive reagent solution is continually supplied during the growth of the nanostructures. The advantages of this method include: (1) directly anchored nanostructures on the desired surface areas without any extra assembly process (no need for doctor-blade or spin coating processes); (2) the reagents (such as the Al source in this work) could be directly delivered to the pre-seeded reaction area which reduces any homogenous nucleation or consumption of the reagents in the bulk solution. Therefore, the morphology and components of the vertically grown nanostructure on indium tin oxide (ITO) coated PET substrate can be readily controlled. ZnO NRs, AZO/ZnO hybrid structures and AZO NFs are obtained vertically, which can meet the critical requirement of a DSSCs photoanode: providing not only highly accessible surfaces but also a long-range electronic connectivity^36^,^37^.

## Results and Disscussion

### Synthesis and characterization of nanostructured photoanode

**Figure 1:**
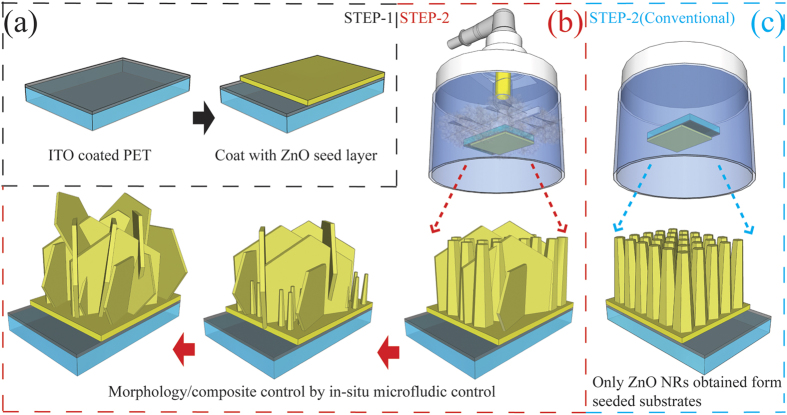
Illustration of the strategies for fabrication of controllable AZO nanostructures (**a**) step 1 for pre-seed substrates; (**b**) step 2 for *in-situ* hydrothermal method used for controllable AZO synthesis; (**c**) step 2 for a reference experiment by conventional hydrothermal synthesis.

**Figure 2:**
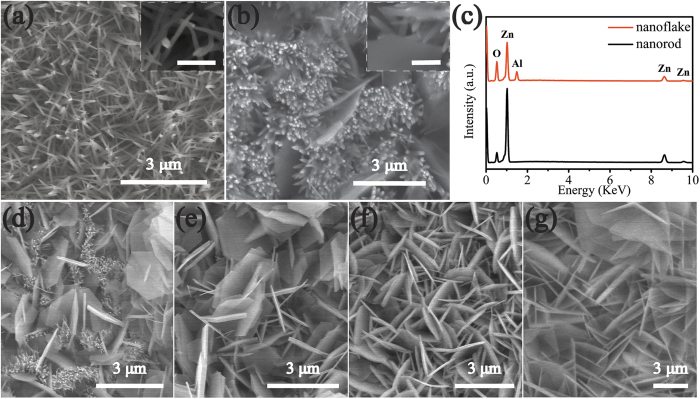
Top view SEM images of (**a**) AZ1-C (1 mM, without MCU) (**b**) AZ1 (1 mM with MCU); (**c**)corresponding EDS of NF and NR in sample AZ1; (**d–g**) the ZnO/AZO nanostructures obtained with MCU by varying Al reactant supply concentration: (**d**) AZ2 (2 mM), (**e**) AZ3 (3 mM), (**f**) AZ4 (4 mM) and (**g**) AZ5 (5 mM). The scale bar in inset of (**a,b**) is 500 nm.

**Figure 3:**
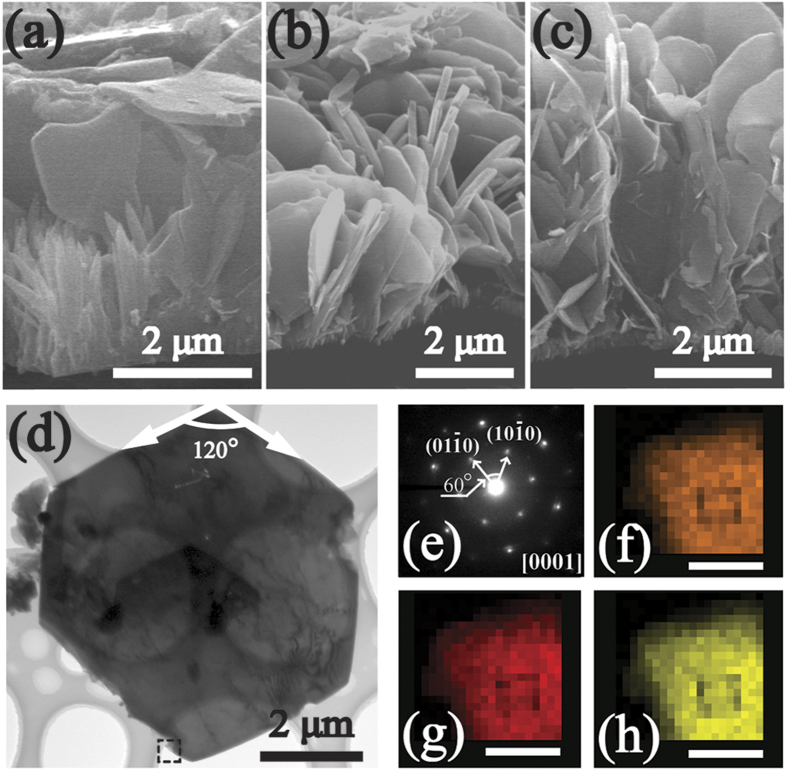
Side-view SEM images of reprezented AZO nanostructures (**a**) AZ1, (**b**) AZ2 and (**c**) AZ4.(**d**) TEM images of a single nanoflake from AZ4; (**e**) The corresponding SAED pattern of AZ4; (**f**–**h**) Element mapping of Zn, Al and O in a selected area (high light by dot line cube in (**d**)) of the nanoflake, scale bar is 100 nm.

Vertically aligned ZnO NRs/AZO NFs hybrid and AZO NFs films were fabricated using the *in-situ* MCU hydrothermal method (refer to the Experimental Section for more details). The morphology and nanostructures can be tuned by varying the supply speeds or concentrations of the additives (i.e., Al reactants in this work, see Fig. 1). The top and side views of the resultant ZnO/AZO nanostructures prepared at various injected concentrations of the additive Al^3+^ sources are shown in Figs. 2 and 3. The control group (AZ1-C, 1 mM) was designed to demonstrate the nanostructures obtained without using the *in-situ* MCU. Using the same chemical solution and reaction conditions as those for the AZ1 samples, only needle-like ZnO NRs can be observed on the pre-seeded substrate (AZ1-C, Fig. 2 (a)). These needle-liked NRs are formed by the face-selective electrostatic control by Al^3+^, which can suppress the sidewall growth^38^. The elemental composition of the NRs was verified using an energy dispersive X-ray spectrometer (EDS) which confirmed that only ZnO NRs were obtained in this condition (Supplementary Fig. S1 (a) online). However, when the *in-situ* MCU was applied in the hydrothermal synthesis with Al^3+^ reactant supply, hybrid nanostructures of ZnO NRs and AZO NFs (Fig. 2(b), AZ1, 1 mM) were obtained. The elemental composition of NRs and NFs was confirmed using EDS analysis as shown in Fig. 2(c) (the corresponding SEM image can be found in Supplementary Fig. S1(b) online). With further increase of the supplied Al reactant concentration (2 to 5 mM), the morphology evolution of the nanostructures is shown in Fig. 2(d–g). Gradually the hexagonal-shaped AZO NFs became dominant and were mostly perpendicular to the substrate surface. Using the lower injected concentrations of Al reactants (i.e., Fig. 2(b), AZ1, 1 mM), the ZnO NRs were dominant in the hybrid structure. With the increase of the injected Al reactant concentrations (2 mM to 3 mM, i.e., AZ2 to AZ3), more and more NRs were replaced by the AZO hexagonal NFs (Fig. 2(d,e)). After the injected Al reactant concentration increased up to 4 mM (AZ4), the AZO NFs showed porous network structures with average pore sizes in a range of 0.3–1 μm. Inter-connected NFs are composed of interweaved plates with a thickness of 70–140 nm as shown in Fig. 2(f). Upon further increasing the injected Al reactant concentration up to 5 mM (AZ5), a porous network structure with larger sizes was observed (Fig. 2(g)). A similar evolution of the film microstructure was observed after varying the injection speeds of Al reagents (see Supplementary Fig. S2 online). Side-view SEM observation of the represented samples revealed that the ZnO NRs and the porous network AZO NFs all started to grow from the seed layer and were mostly perpendicular to the seed layer. Figure 3(a) clearly reveals that the AZ1 hybrid nanostructures are a mixture of needle-like ZnO NRs and flake-like AZO. As shown in Fig. 3(b), the AZ2 sample has vertically aligned flake nanostructures on the substrate with an average lateral size of ~3.9 to 4.7 μm. When the injection concentration was increased to 4 mM (i.e., sample of AZ4), a network morphology of the NFs can be observed as shown in Fig. 3(c). Further increasing the reaction concentration up to 5 mM, the network morphology of the NFs is found to connect at the roots (See Supplementary Fig. S3 online). This may be due to the increased growth rate caused by a higher injected concentration and size increase of the NFs as they grow larger (Fig. 2(g)), which is commonly reported for growth of ZnO nanostructures using the solution based process^39^,^40^. The formation of the NFs can be further confirmed from the TEM image shown in Fig. 3(d) which reveals a single NF was obtained from AZ4 with a hexagonal morphology. The corresponding electron diffraction pattern is shown in Fig. 3(e) for the AZ4 NF along the [0001] zone axis, which is parallel to the vertical direction of its top-side. Clearly it indicates the single crystalline wurtzite structure of an AZO NF. The single crystalline structure of the NF could also be verified from the XRD results shown in Supplementary Fig. S4 online. Qualitative analysis of the AZ4 NF sample was carried out using the scanning transmission electron microscopy mode together with the EDS analysis. Results shown in the Fig. 3(f–h) clearly reveal that Zn, Al and O elements are homogeneously distributed inside the NFs.

Evolution of the resultant nanostructures can be explained using both “competing reaction” and “heterogeneous nucleation growth” mechanisms^29^,^39^. The solution-phase ZnO precursor (i.e. Zn(OH)_n_^2−n^, Zn(NH_3_)_n_^2+^, n = 1,2,3,4) can either react with Al(OH)_4_^−^ to form AZO or react with OH^−^ to form ZnO under suitable conditions. For the pH range of the solution used in this experiment, the solution-phase ZnO precursor reacts preferentially with the Al complex (i.e. Al(OH)_4_^−^, etc.) because it is thermodynamically favored^29^,^41^. Using this *in-situ* MCU, the Al reactant can be delivered directly to the reaction zone, thus avoiding the possibility that most of Al reactants would be consumed by a homogeneous nucleation in the bulk solution. Therefore, with the increase of the amount of Al reactant injected to the reaction area (i.e., the solution surrounding the seeded substrate), the ZnO NRs grown from the seed-layer gradually change into the AZO NFs.

**Figure 4:**
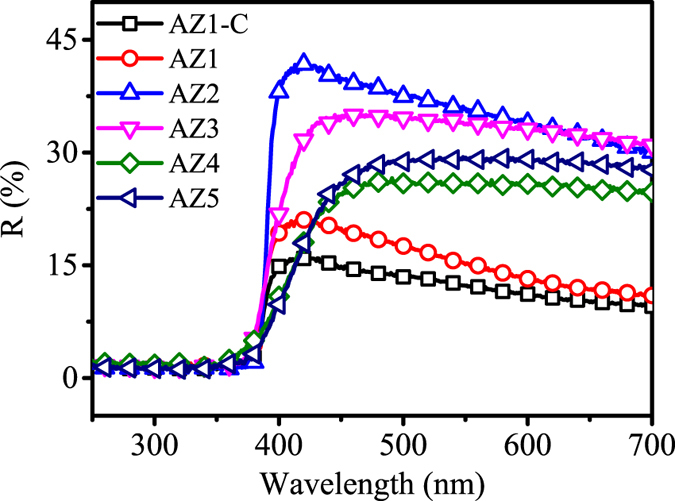
Diffuse reflectance spectra of the AZ1-C and AZ1 to AZ5 photoanode.

Before assembling the nanostructured photoanodes into DSSCs, the reflectivity of each sample was investigated for its scattering effect of the nanostructure. Figure 4 shows the diffuse reflectance UV-Vis spectra obtained from ZnO and AZO photo-anodes before the dye adsorption. Apparently, insignificant reflectivity is observed in the UV range for all the samples, which can be attributed to the strong UV absorption of wide-band gap ZnO nature^42^. The AZO photoanodes (AZ1 to AZ5) show higher reflectivity values in the wavelengths from 400 to 700 nm than those of the pure ZnO NRs (AZ1-C). The intensity of the reflection was increased from ZnO NRs (AZ1-C), AZ1, and then to AZ2, which can be explained from the increased light scattering caused by a combination of hybrid nanostructures of NRs and nano-flakes^43^,^44^. There is a decreasing trend in the intensities of the reflection from samples of AZ2 to AZ5, probably because the increased size of the AZO NFs (compare to NRs) could improve light trapping^22^. As suggested by Peng *et al.* based on their simulation using finite difference time domain and experimental verification, the NFs with small sizes can cause a random light scattering. Once the NFs have grown to sizes above 2 μm, the randomly distributed directions of the NFs will greatly improve the light trapping effects^22^. This result confirms that the light scattering effect did occur in the nanostructures.

### Electrical properties of DSSCs

**Figure 5:**
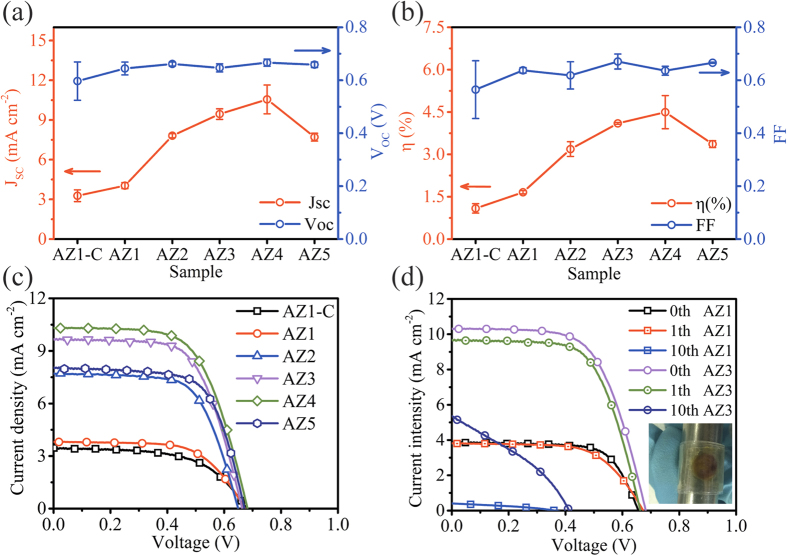
J-V characteristics of DSSCs assembled by nanostructured photoanode prepared under different Al reactant injection concentration. (**a,b**) The parameters of DSSCs extracted form J-V measurement. (**c**) The represented J-V curves of different structured photoanode under simulated AM1.5, 100 mW cm^-2^ solar irradiation. (**d**) J-V curves of DSSCs made by AZ1 and AZ3 after 1th and 10th cycle bending at bending radius of 10 mm. Inset: photography by wrapping the DSSCs around a 10 mm diameter steel rod.

The nanostructured photoanodes were assembled into the DSSCs and key performance parameters of the solar cell samples have been extracted from current density vs. voltage (J-V) curves obtained under the simulated sunlight as shown in Fig. 5(a,b). Clearly, there is a significant enhancement of the performance after the introduction of AZO NFs. The DSSCs based on pure ZnO NRs (AZ1-C) and hybrid ZnO NRs/AZO NFs (AZ1) show average conversion efficiencies of 1.09% (J_sc_ = 3.27 mA cm^−2^, V_oc_ = 0.59 V, FF = 0.56) and 1.66% (J_sc_ = 4.04 mA cm^−2^, V_oc_ = 0.64 V, FF = 0.64), respectively. With increasing Al reactant injection concentration up to 2 mM (AZ2) during the nanostructure growth, both the values of J_sc_ and V_oc_ increased sharply with typical values of 7.82 mA cm^−2^ and 0.66 V, respectively, achieving a PCE of 3.19%. With further increase of the injection concentration (i.e., AZ3 and AZ4, which were grown from higher Al reactant injection concentration, 3 mM to 4 mM), the performance parameters of the DSSCs were further improved. These DSSCs based on the AZO NF dominant samples (AZ3 and AZ4) showed average conversion efficiencies of 4.10% and 4.50%, respectively. However, the DSSC based on AZ5, which was obtained with a higher Al reactant injection concentration up to 5 mM, has an average PCE of 3.37% (J_sc_ = 7.71 mA cm^−2^, V_oc_ = 0.66 V, FF = 0.67).

Cyclic bending tests were performed under different bending cycles with a maximum bending angle of −180° to +180° (radius = 10 mm, Supplementary Fig. S5 online) for these DSSCs made on PET substrates. Figure 5(d) shows the J-V characteristics of devices AZ1 and AZ3 under different bending cycles. It can be noticed that no significant degradation in J-V performance of the DSSCs was observed after the cyclic bending. Recently, novel designs of flexible DSSCs were investigated in order to improve their performance (e.g. using new materials of transparent conducting film or planar-structure design of electrodes)^45^,^46^. According to those studies, the mesoporous material layer was vulnerable to cracking and spallation, after even one bending cycle, and the performance of the devices was dramatically deteriorated. However, in this study, the performance of the DSSCs did not show apparent deterioration under +180° to −180° cyclic bending at a radius of 10 mm. This suggests that the hybrid nanostructures indeed have good adhesion to the ITO coated flexible substrates and high resistance to cracking due to their anchoring effects which benefit from the directly-grown process. However, as shown in Fig. 5(d), after bending for 10 cycles, the value of J_sc_ of the AZ1 was decreased from 3.81 to 0.40 mA·cm^−2^, and the value of V_oc_ was decreased from 0.67 to 0.36 V. For the sample of AZ3, the value of J_sc_ was decreased from 9.68 to 5.33 mA cm^−2^, and the value of V_oc_ was decreased from 0.66 to 0.41 V. During cyclic bending, the nanostructured layer experienced significant repeated tensile/compressive strains, which apparently caused the fracture/cracking of the nanostructured photo-anode thus degrading the performance of the device^45^,^46^. Furthermore, the degradation of ITO electrodes during cyclic bending (Supplementary Fig. S6 online) might also degrade the electrical properties of the cell. This could be improved by using flexible transparent conducting material (e.g. graphene) and/or novel planar-structured electrodes or double mesh electrode to replace the conventional sandwiched solar cell structure^45^,^46^,^47^.

**Figure 6:**
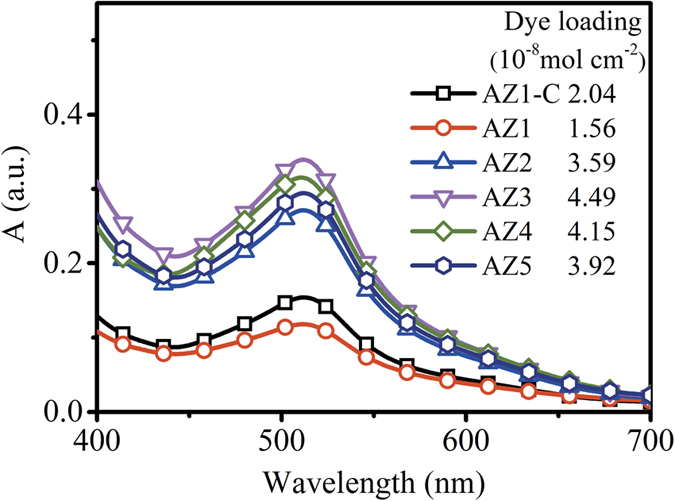
Absorption spectra of dye desorbed from AZ1-C and AZ1 to AZ5 films with an area of 1.6 cm^2^. The insert table is dye loading of corresponding samples.

To gain a better insight into the improvement of the DSSCs’ performance, dye loadings of all the DSSC samples using the solution containing dye desorbed from each sample were compared based on the measurement results from the UV–Vis spectrophotometer. Figure 6 shows the optical absorption spectra obtained from the solutions desorbed from various samples. Compared with the pure ZnO NRs (AZ1-C), there was an increasing trend in dye-loading capabilities of the AZO samples with increasing Al injection concentration. This is mainly because the increased internal surface areas (due to the formation of network porous structures of the AZO NFs) result in an increased dye loading, thus an increased efficiency.

However, compared with the ZnO NRs sample (AZ1-C), the sample AZ1 did not show an apparent increase in dye loading like the other AZO samples. This is probably attributed to the reduced dye-accessible areas of the AZ1 hybrid nanostructures, which could be caused by (1) larger sizes of the NFs (compared to the NRs in AZ1) grown among bunches of NRs which could block the original porous spaces between a group of NRs; (2) the amount of the NFs in AZ1 is still low and has not yet achieved a porous network structure in this sample^48^,^49^. Although dye loading in the AZ1 hybrid structures was lower than that of the AZ1-C, all the key parameters of the DSSCs based on the AZ1 sample have been improved. Additionally, a similar trend was also observed in the groups of AZ3 and AZ4. Though dye loading of AZ4 was slightly lower than that in the AZ3, all the other key parameters of the DSSCs based on sample AZ4 were improved (J_sc_ = 10.55 mA cm^−2^, V_oc_ = 0.67 V, FF = 0.64), resulting in an increased PCE value of 4.50%. It is noticeable that the AZ5 device showed decreased J_sc_ and PCE values but a relatively high V_oc_ value. This could be attributed to the significant decrease in dye loading in the sample of AZ5 (as shown in Fig. 6) which was caused by the reduction of surface areas because of the connections of the roots of the nanostructures^40^. Bai *et al.* also reported the enhancement of all the key parameters of the DSSCs while dye loadings were decreased for a photo-anode made from hybrid nanostructures of ZnO nanowires network within TiO_2_ nanoparticle films^49^. They found that proper amount of ZnO NRs embedded inside TiO_2_ nanoparticle films could improve the overall performance of DSSCs although dye loading was reduced, due to the retardation of electron-recombination losses by improved electron mobility of the hybrid film^50^,^51^. It is worth noting that for the samples AZ3 and AZ4, solar cell performance, particularly the values of V_oc_ and J_sc_, were increased despite the slightly lower amount of adsorbed dye. Specifically, the V_oc_ value of the AZ4 is larger than that of the AZ3, which should be attributed to the suppressed electron-recombination in these devices. Therefore, it can be predicted that the performance of the DSSCs can be enhanced further by improving dye loading ability of AZO NFs based photoanodes. Normally, for the 1&2D nanostructures, the longer nanorods (or the thicker film) could improve dye loading and thus improve the overall DSSC performance. Accordingly, the DSSCs based on the Al reactant injection concentration of 4 mM has been chosen to study the film thickness effect (details can be found in Supplementary Fig. S7 online). The film thickness increased with growth durations, however, the dye loading capability does not increase with film thickness (Fig. S7(a)) in this study. This is because the nanostructures became coalesced after a longer growth duration (Fig. S7(c,d)). As a result, the best performance was achieved with the DSSC sample of AZ4 (after 4 hrs growth), with the highest dye loading in this study.

**Figure 7:**
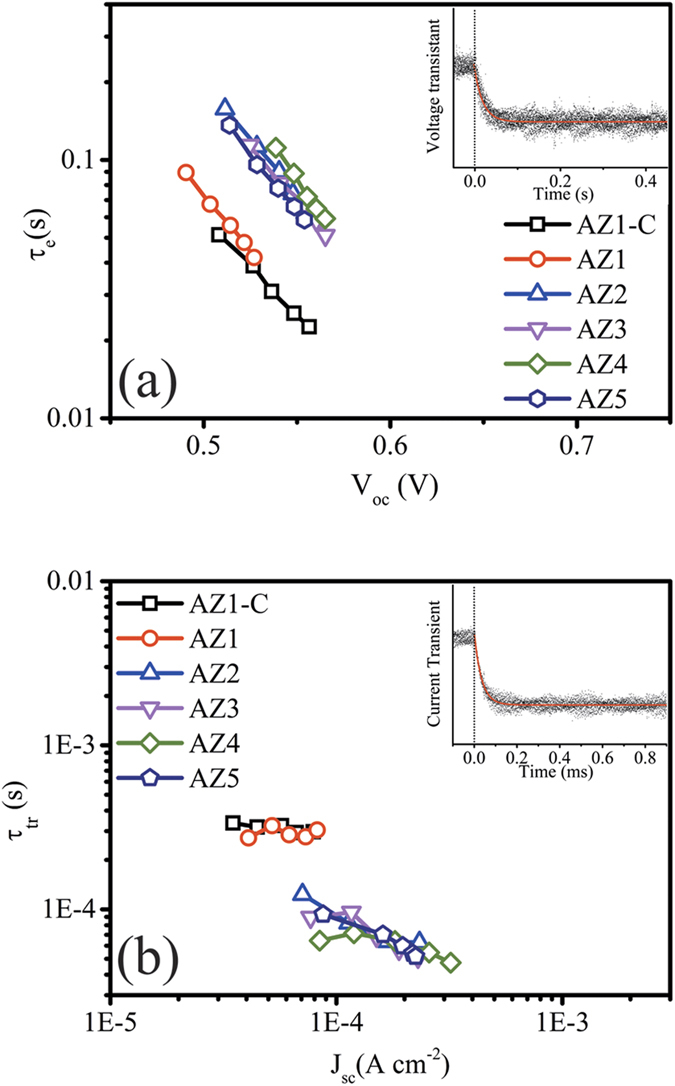
Electron transport and recombination kinetics for DSSCs made by different photoanode (AZ1-C, AZ1 to AZ5): (**a**) Electron life time constant extracted from devices with varying pulse light intensity. (**b**) Electron transport constant which estimated from current transient decay obtained under different pulse intensity. Insert in each image is an example of voltage-transient/current transient with an exponential decay fit, respectively.

The reduced recombination can be confirmed by investigating the dark current data (as shown in Supplementary Fig. S8 online). It is generally agreed that, in the dark condition, the potential distribution across the cell is different from that under illumination, which can be used to estimate the extent of the back electron transfer^49^. In Fig. S8, the dark current of the as-prepared DSSC (from AZ1-C, AZ1 to AZ5) shows a shift to a higher potential value and the dark current becomes smaller at the same potential (i.e., 0.6 V). The charge carrier lifetime (τ_e,_ recombination time) has been extracted from voltage decay measurements as shown in Fig. 7(a). The inset in Fig. 7(a) shows a representative result of transient decay in the V_oc_ values as a function of time based on fitting using single exponential decay^52^. The τ_e_ values of different samples obtained over a range of light intensities are plotted against the corresponding V_oc_ value as shown in Fig. 7(a). It is noticed that the τ_e_ values show a similar trend to that of the V_oc_ results as shown in Fig. 5(a), and τ_e_ values are shifted to larger values with increased Al reactant injection concentrations, demonstrating that the electron-recombination process was retarded. Also, the τ_e_ values are shifted significantly to larger values once the AZO NFs become dominated (AZ2 to AZ5). According to previous research, n-n^ + ^heterojunction could be formed between the ZnO based nanostructures and TiO_2_ modification layers which would bring a build-in potential between them, thus suppressing the recombination. This build-in potential is proportional to the ratio of electron concentration between ZnO/AZO based nanostructures and TiO_2_ modification layers^18^,^53^,^54^. In this case, AZO has a higher electron concentration than ZnO, thus the increased ratio of electron concentration between ZnO/AZO based nanostructure and TiO_2_ modification layer results in a higher build-in potential, which further enhances the suppress of recombination.

To verify the assumption that the AZO NFs could improve the electron mobility of the photoanode, the current transient measurements at short circuit conditions for the DSSCs made by AZ1-C to AZ5 photoanode were performed. Each transient result was fitted by a single exponential decay (as shown in the inset of Fig. 7(b) for one representative example). The electron transport time constant (τ_tr_) of different samples obtained over a range of light intensities are plotted against the corresponding J_sc_ values as shown in Fig. 7(b). The samples of AZ2 to AZ5 did not show significant differences in the time of electron transport (τ_tr_), however, their time constants are significantly lower than those of the AZ1-C and AZ1. Again, the AZO NFs dominated sample group shows a significant shift of electron transport time when compared with those of the ZnO-dominated samples. Consequently, the enhanced PCE of the AZO NFs dominated samples (AZ2 to AZ5) can be attributed to the network structures with less grain boundaries and defects, thus realising the suppressed recombination for a fast electron transport, longer electron lifetime^55^. Similar trends have also been observed in AZO nanorod based DSSCs by Sining *et al.*^56^, and they reported the Al doped ZnO NRs showed a remarkable enhancement of performance because of their increased electrical conductivity compare to those of the pure ZnO NRs.

## Conclusion

We have developed an *in-situ* MCU in a hydrothermal synthesis process to achieve a controllable approach to obtain AZO nanoflakes and ZnO/AZO hybrid structures directly on flexible polymer substrates. Results from TEM, EDS and XRD analysis confirm that the morphology and composition of the nanostructure can be readily changed from crystalline ZnO NRs, to a hybrid structures of ZnO NRs/AZO NFs, and finally to AZO NFs. We then demonstrated that flexible dye-sensitized solar cells (DSSCs) can be fabricated based on these nanostructures, and diffuse reflectance spectra suggested that the light-trapping efficiency of the AZO nanostructures was improved over those of the pure ZnO NRs due to an effective light-scattering caused by the nanoflake and hybrid nanostructures. The AZO NFs vertically aligned on the flexible substrates improved the accessible surfaces (i.e. benefit from improving dye loading) with a demand for a long range electronic connectivity (i.e. benefit for reducing recombination). As a result, the power conversion efficiency of the AZO-based DSSCs has been improved. The current transient study indicated that electron transport property of AZO samples has been improved and recombination in the AZO dominated samples has been suppressed. This i*n-situ* MCU hydrothermal method is promising for low cost, low temperature and efficient fabrication of hybrid nanostructures due to its simple setup and reliability.

## Experimental

### Preparation of Nanostructured Photoanodes

All chemicals used in this study were purchased from Sigma Aldrich and used as received. The ITO coated PET substrates were obtained from Solaronix (PETITO175-14). ZnO seed layer, 50 nm thick, was deposited on the PET substrates using a DC magnetron sputter with a pure Zn target (99.99 at%) in an Ar (15 sccm) and O_2_ (15 sccm) mixture and a pressure of 5 mTorr. The hydrothermal growth condition was chosen based on a predicted model (Fig. S9)^38^ and ref. 29. The basic solution was 100 ml transparent aqueous solution containing 25 mM zinc nitrate hexahydrate (Zn(NO_3_)_2_·6H_2_O, ≥ 99.0%) and 400 mM ammonium chloride (NH_4_Cl, ≥ 99.5%) with a pH value of 10.8 (adjusted by sodium hydroxide). The substrate was placed upside down in the solution in a sealed reaction vessel, connected with a syringe pump (see Fig. 1(b)). All the synthesis was performed at 80 °C by placing the reaction vessel into a pre-heated water bath. During the film growth, aluminum nitrate nonahydrate (Al(NO_3_)_3_·9H_2_O, ACS reagent, ≥ 98.5%,) with final total concentrations of 1 mM to 4 mM was injected into the solution through the syringe pump, and control of the nanostructures was achieved by varying the injection speeds and concentrations. The concentrations of the solutions used for injection at a speed of 2 mL/hr were set at 1, 2, 3, 4 and 5 mM, which were denoted as samples AZ1, AZ2, AZ3, AZ4 and AZ5, respectively. The injection speeds were varied between 2 mL/hr to 4 mL/hr with a total injected final content of 2 mM, which were denoted as samples 2 mL/hr-AZ1S, 3 mL/hr-AZ2S and 4 mL/hr-AZ3S (Supplementary Fig. S2 online). The duration of the reaction was fixed at four hours. One control group was set to investigate the *in-situ* MCU effects; one sample (AZ1-C) was prepared using a standard hydrothermal method using the same chemical solution and reaction conditions as AZ1 but without *in-situ* microfluidic unit. For thickness effect experiments, the durations of the growth were varied from 1 to 12 hrs (1, 2, 4, 8, 12) with 4 mM of the solution used for injection at a speed of 2 mL/hr, which were named as AZ4-1 hr, AZ4-2 hr, AZ4, AZ4-8 hr, AZ4-12 hr, respectively. After synthesis, all the samples were rinsed with DI water and dried with N_2_ gas. A layer of anatase TiO_2_ (~55 nm) was then deposited on the as-prepared samples using a low temperature deposition process reported in our previous study^57^.

### Characterization of Nanostructured Films

Surface and cross-section morphologies of the samples were characterized using scanning electron microscopy (SEM, S-4100 Hitachi) and transmission electron microscopy (TEM, JEOL JEM-2100). The thickness of the nanostructured film had been measured by stylus profilometry (Dektak XT, BRUKER) and SEM. Photoanodes with sub-wavelength structures (i.e., nanowire, nanotube and multi-sized particle based porous structure) have been reported to increase the light trapping effect via scattering effects^58^,^59^. Therefore, light scattering properties of the samples were measured using a light absorption/diffuse reflectance spectrometer with an integrating sphere (Perkin-Elmer Lambda 9).

### Fabrication and Measurements of DSSCs

The resultant AZO photoanodes were sensitized by immersing the devices into a solution of N719 dye (0.3 mM in MeCN/t-Butanol, 1:1) in a dark environment for 10 hours. The samples were then rinsed with the same (1:1, MeCN/t-Butanol) solvent to remove excess physically adsorbed dye. A Pt layer of ~5 nm thickness was sputter-deposited on the ITO coated PET as the counter electrode. The sensitized photoanode and counter electrode were sandwiched using a 50 μm thick Surlyn sealing frame (MS004620, DYESOL) with an active area of ~0.28 cm^2^. The internal gap between the electrodes was filled with iodide/triiodide (I^−^/I_3_^−^) electrolyte (0.1 M LiI, 0.1 M I_2_, 0.5 M tert-butylpyridene, and 0.6 M tetrabutylammonium iodide in acetonitrile). The results of photo current density-voltage (J-V) were obtained using AM 1.5G simulated sunlight irradiation (100 mW·cm^−2^) (Sciencetech Inc. SF150) and an electrochemical instrument (PGSTAT302N, Auto lab) with a black mask to define the active area of the cells. A standard solution (0.1 M NaOH in ethanol-water (1:1)) was used to perform dye-desorption, and the corresponding dye loading was estimated by UV–Vis spectrophotometer (UV-365, Hitachi)^60^.

The electron kinetics in the flexible DSSCs was investigated by measuring the transient electrical signals (voltage/current) after optical perturbation. Transient photocurrent and photovoltage decay were taken based on a function generator (33120A, Agilent) and a white light emitting diode (LED)^61^. The time resolved voltage and current measurements were recorded using an oscilloscope (MSO-X, 3054A, Agilent). The current was determined by Ohm’s Law from measuring the voltage drop across a measurement resistor (36 Ω) which was operated in series with the DSSC. Multi-runs were performed for noise reduction.

## Additional Information

**How to cite this article**: Zhao, C. *et al.* *In-situ* microfluidic controlled, low temperature hydrothermal growth of nanoflakes for dye-sensitized solar cells. *Sci. Rep.* **5**, 17750; doi: 10.1038/srep17750 (2015).

## Supplementary Material

Supplementary Information
