# Supplementary material for: In-situ microfluidic controlled, low temperature hydrothermal growth of nanoflakes for dye-sensitized solar cells
Source: Sci Rep. 2015 Dec 3;5:17750. doi: 10.1038/srep17750 (PMC4668546; doi:10.1038/srep17750)
Supplement: Supplementary Information [file srep17750-s1.pdf]

Supplementary Information

In-situ **microfluidic controlled, low temperature hydrothermal growth of nanoflakes for dye-sensitized solar cells**

Chao Zhao[1,2], Jia Zhang[3], Yue Hu[4], Neil Robertson[4], Ping An Hu[3], David Child[5], Desmond Gibson[5], Yong Qing Fu[1,2] *

[1] Faculty of Engineering & Environment, Northumbria University, Newcastle upon Tyne, NE1 8ST, UK

[2] Thin Film Centre, Scottish Universities Physics Alliance (SUPA), University of the West of Scotland, Paisley, PA1 2BE, UK

[3] Key Lab of Microsystem and Microstructure, Harbin Institute of Technology, Ministry of Education, No. 2 YiKuang Street, Harbin, P.R. China, 150080

[4] University of Edinburgh, Joseph Black Building, David Brewster Road, Edinburgh, EH9 3FJ, UK

[5] Institute of Thin Films, Sensors & Imaging, University of the West of Scotland, Paisley, Scottish Universities Physics Alliance, PA1 2BE, UK

Corresponding author:

Department of Physics and Electrical Engineering
Northumbria University, Newcastle upon Tyne, NE1 8ST, UK
Email address: richard.fu@northumbria.ac.uk

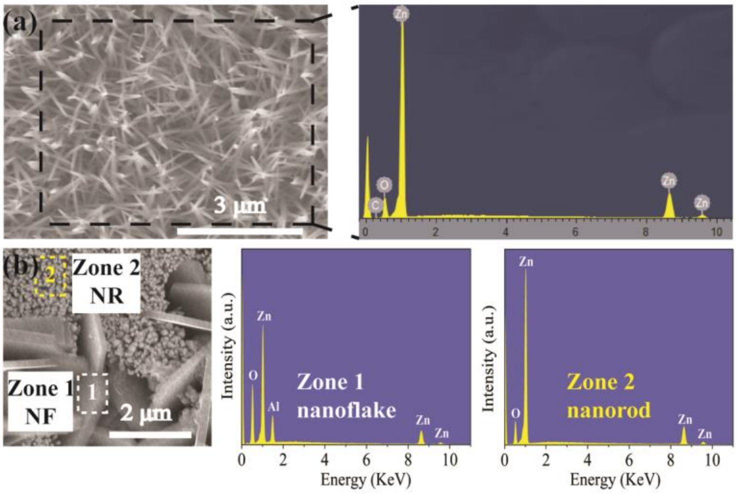

Figure S1. Top view SEM images of (a) the needle-liked ZnO NRs obtained in the control group experiment (AZ1-C, 1 mM) with its corresponding EDS spectra took from selected area; (b) AZ1 (1mM with MCU) with its corresponding EDS of selected area for nanoflake and nanorod, respectively

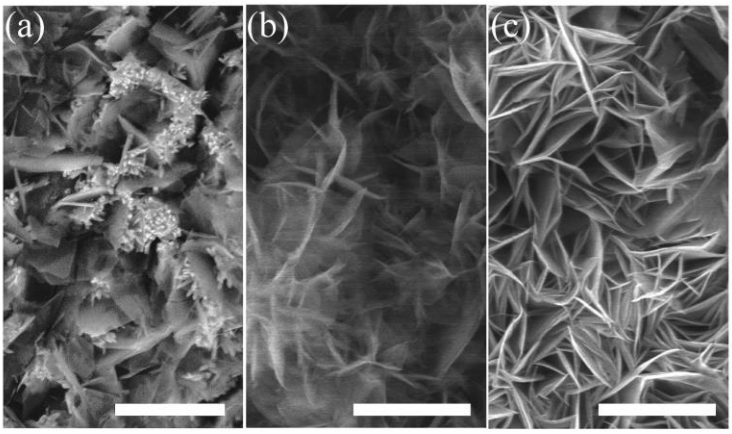

Figure S2. SEM images of AZO NFs films grown on ITO coated PET substrates with different injection speeds (a) 2 ml/hr-AZ1S (b) 3 ml/hr-AZ2S (c) 4 ml/hr-AZ3S. Scale bar in each image is 3 μm.

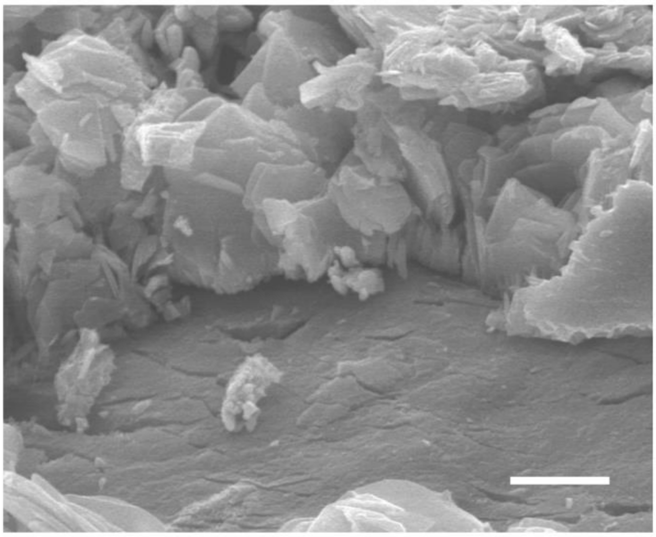

Figure S3. Side-view SEM image of AZ5 nanostructures. Scale bar is 3 μm

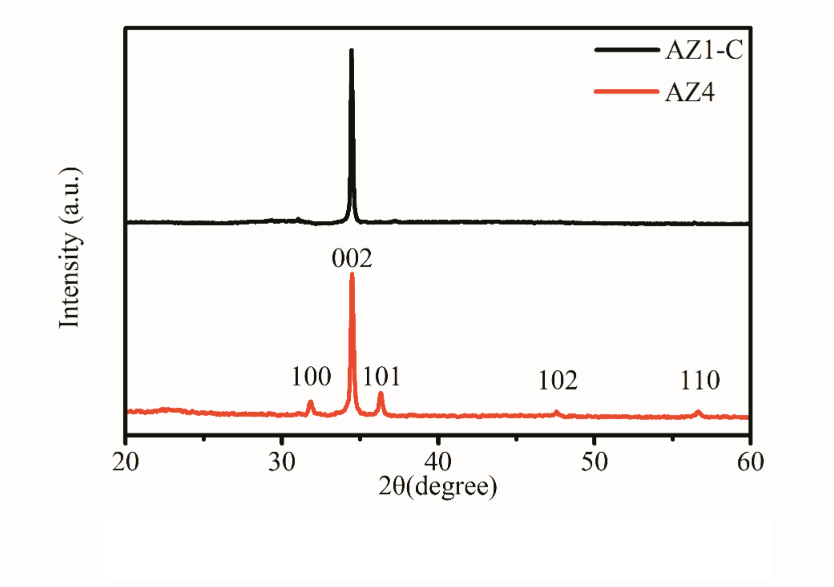

Figure S4. XRD patterns of sample AZ1-C and AZ4 on PET substrates.

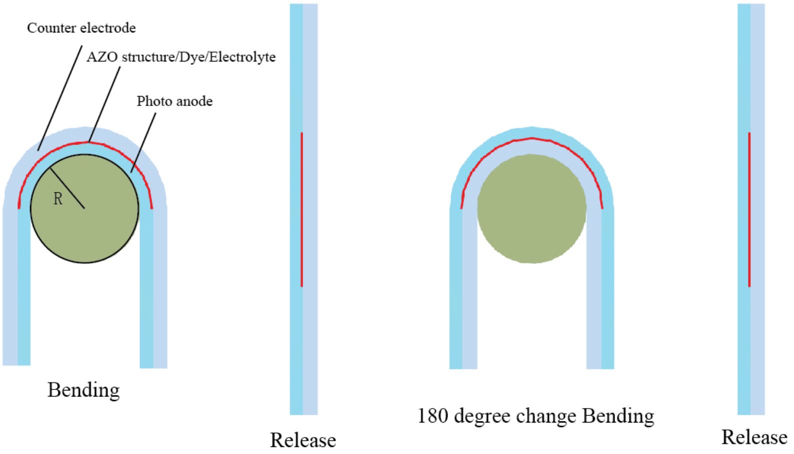

Figure S5. One complete bending test cycle : stretch-release-compression-release.

The flexable DSSCs were bent from -180° to +180° (radius, R=10 mm) with a bending speed at 0.1 Hz.

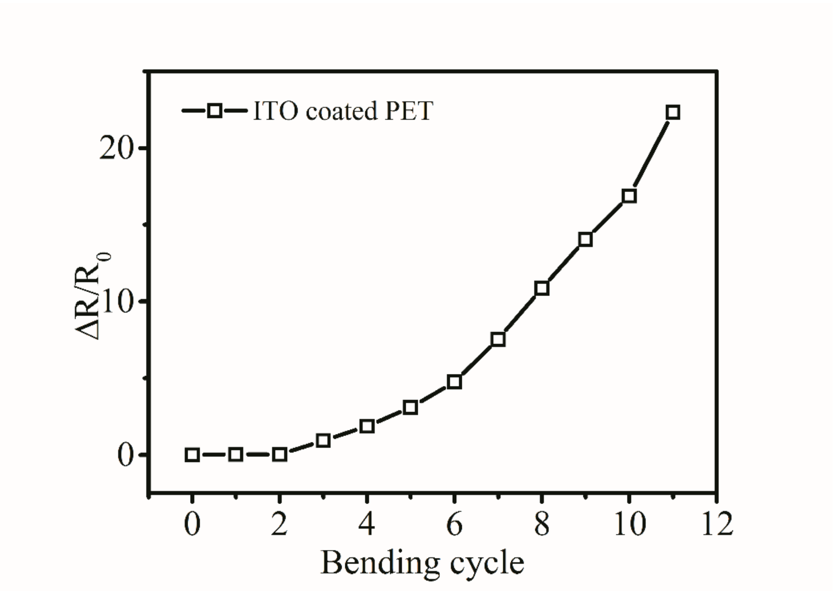

Figure S6. Electrical conductivities of ITO-PET under different -180° to +180° bending cycles. R0 and ΔR are correspondent to the square resistance of the ITO-PET substrates before and after bending.

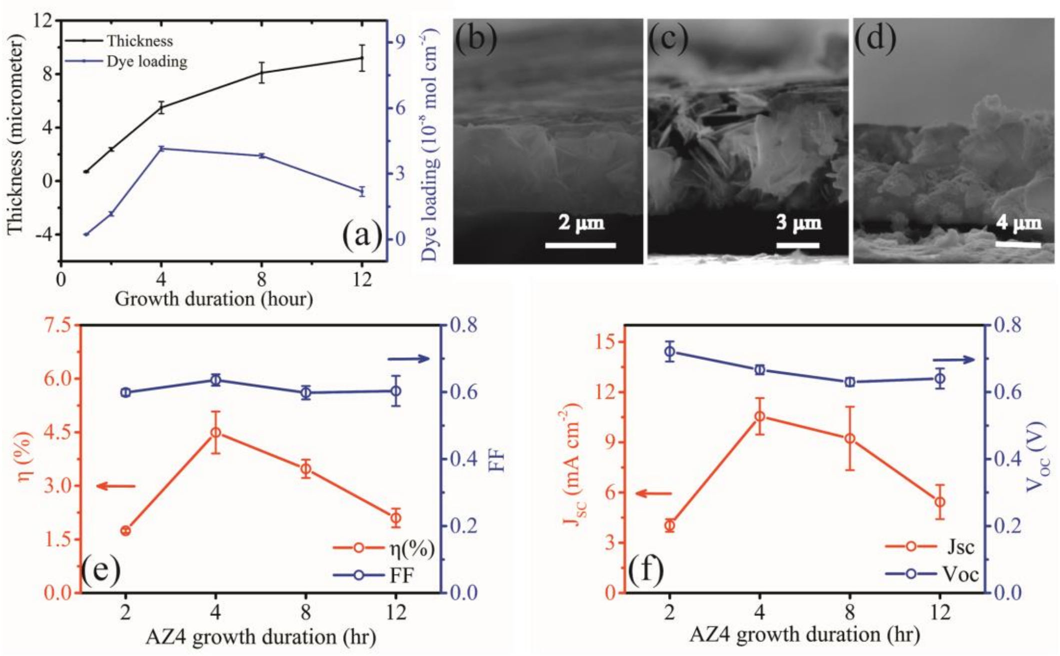

Figure S7. Dependences of the DSSCs on the growth duration under the injection concetration set to 4 mM. (a) film thickness plot as a function of growth duration; (b) to (d) represent cross-section images of sampel (b) AZ4-2hr (c) AZ4-8hr (d) AZ4-12hr , (e) PCE and FF (f) Jsc and Voc of DSSCs based on AZO nanostructured photoanodes parepared under different growth durations.

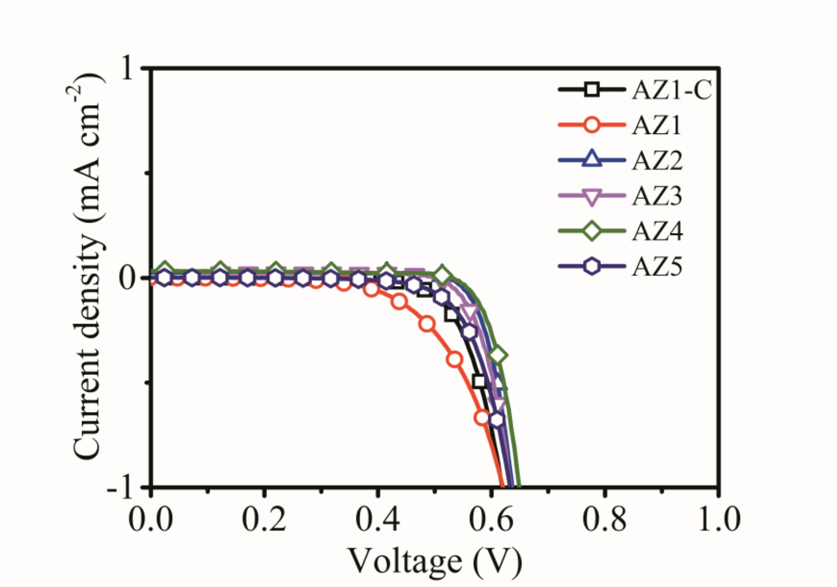

Figure S8. J-V characteristics of DSSCs based on nanostructured photoanodes prepared under different injection concentrations in a dark condition.

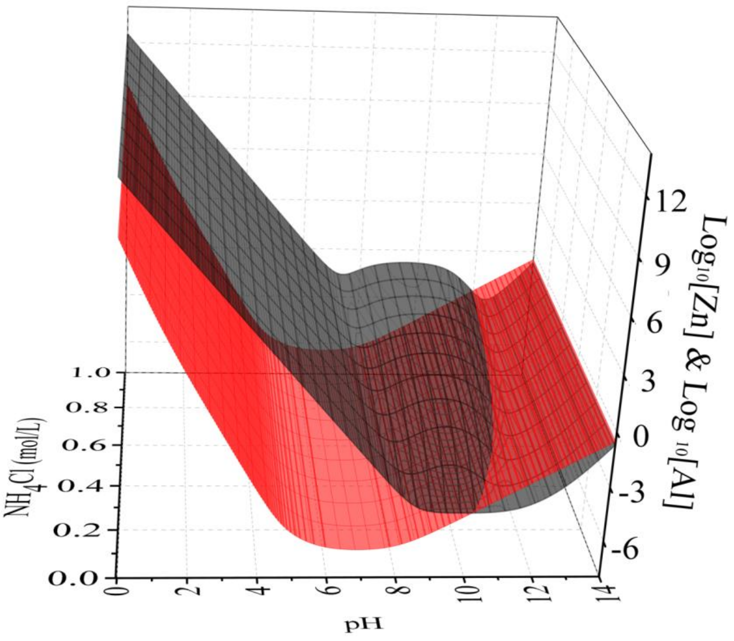

Figure S9. Solubility plots of Zn reactant (black) and Al (red) reactant with different pH/NH_4Cl concentrations predicted using the model proposed in Ref.[38]

It shows solubility plots calculated from the possible chemical reactions in the ZnO nanostructure growth solution with Al source additives based on the model proposed by Joo et al [38]. It descripts a maximum concentration of Zinc & Al ions with/without forming oxide by varying pH and $NH4^+$ concentrations. For solutions with composition below the 3D contour plane shown in the Fig S9, no precipitation or nanostructure growth will occur.
